# Supplementary material for: Structural insight into the function of human peptidyl arginine deiminase 6
Source: Comput Struct Biotechnol J. 2024 Aug 16;23:3258–69. doi: 10.1016/j.csbj.2024.08.019 (PMC11402830; doi:10.1016/j.csbj.2024.08.019)
Supplement: Supplementary file 4 — Supplementary material [file mmc4.docx]

**S1B Fig – Raw gel:**

**
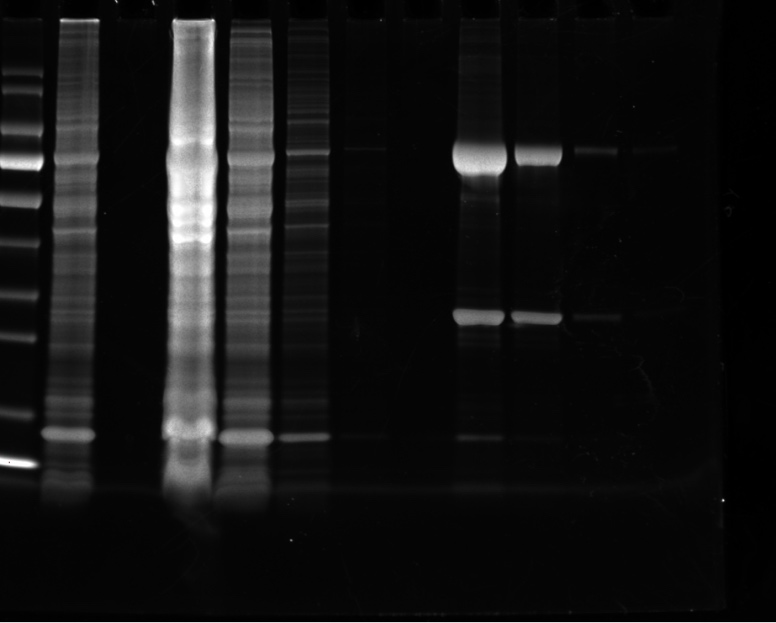
**

**S1C Fig – Raw gel:**

**
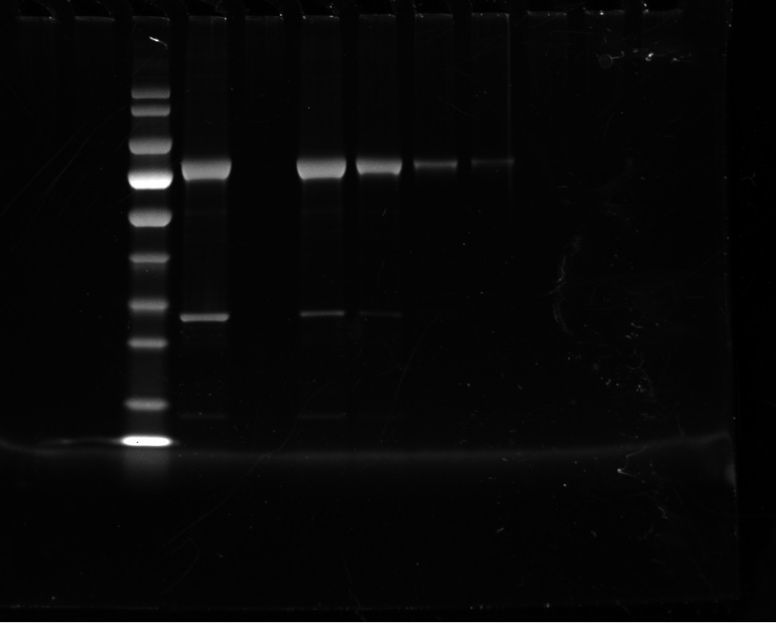
**

**S1E Fig – Raw gel:**

**
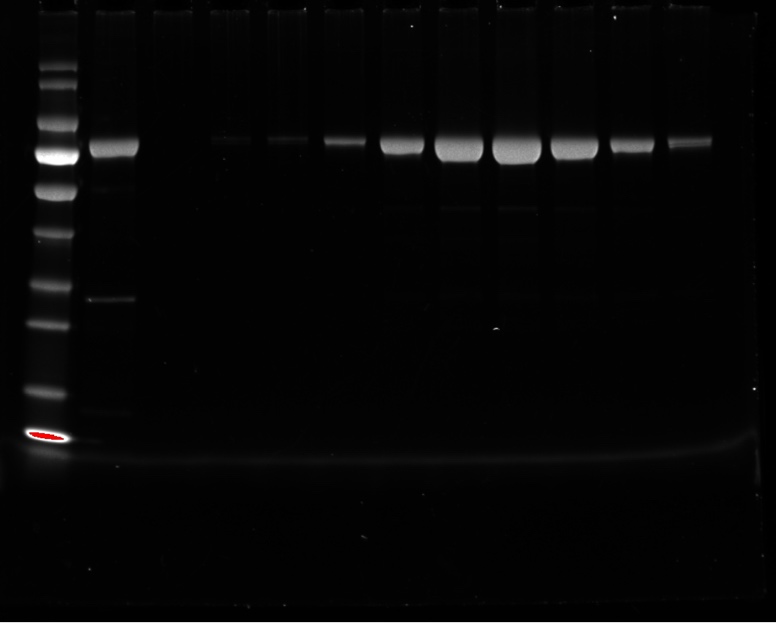
**

**S8 Fig – Raw gel: PADI6**

**
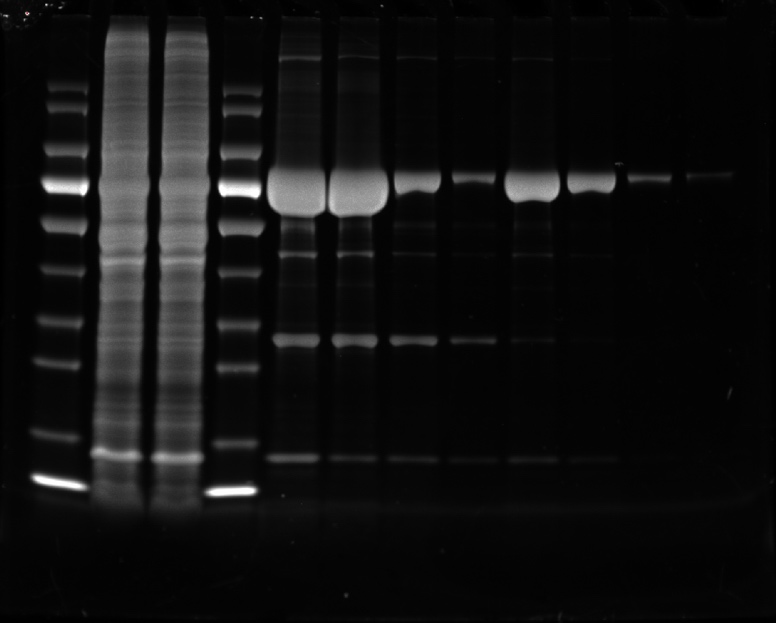
**

**S8 Fig – Raw gel: PADI6^N598A^**

**
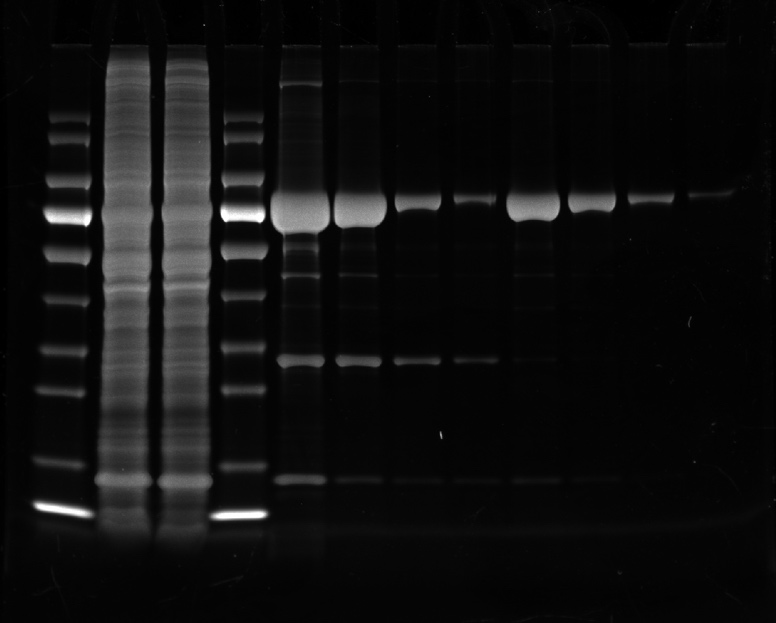
**

**S8 Fig – Raw gel: PADI6^E670A, D673A^**

**
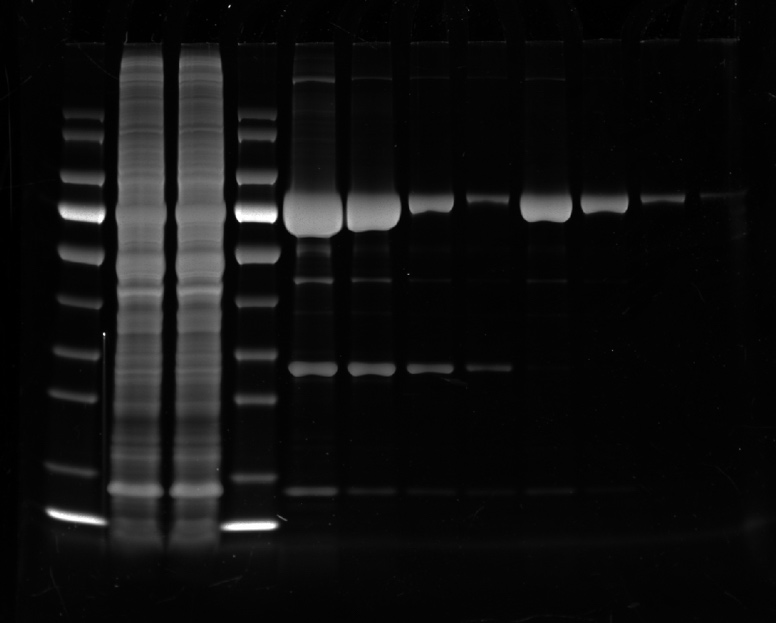
**

**S8 Fig – Raw gel: PADI6^R355A^**

**
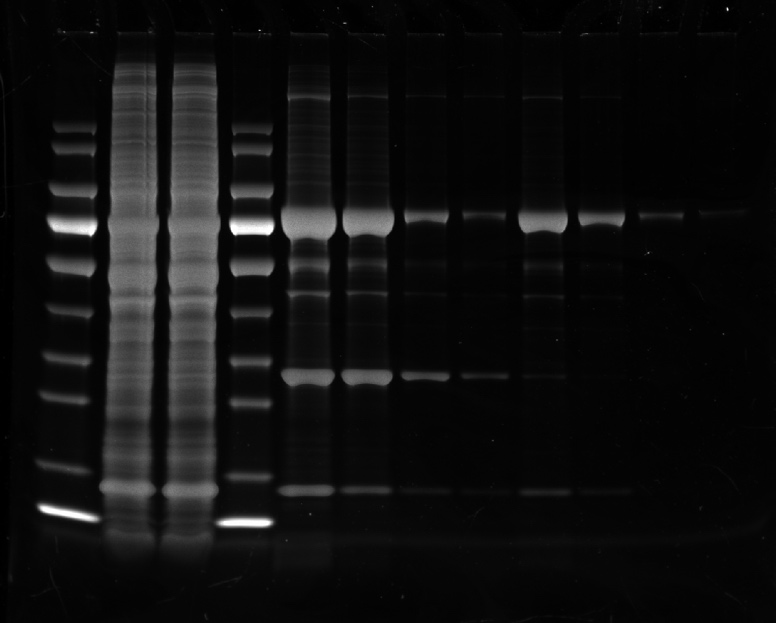
**

**S8 Fig – Raw gel: PADI6^R355A, N598A^**

**^
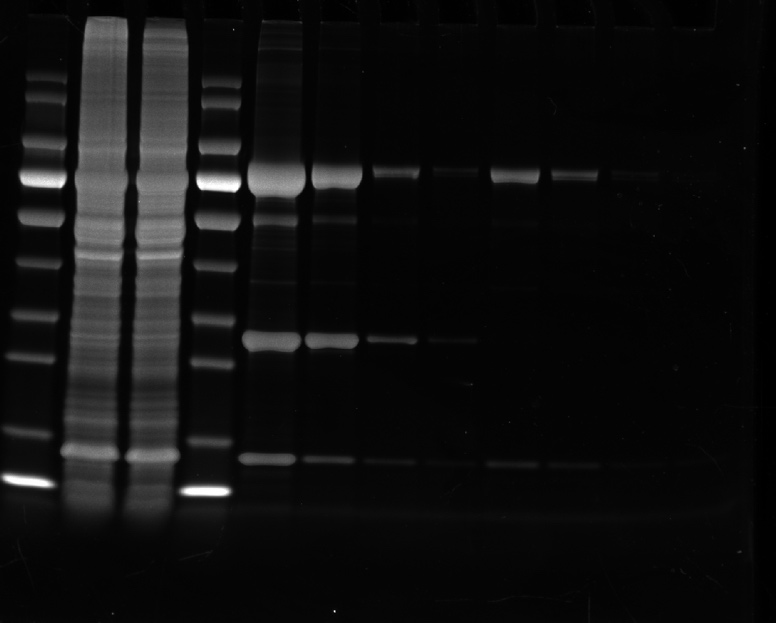
^**

**S8 Fig – Raw gel: PADI6^R355A, E670A, D673A^**

**
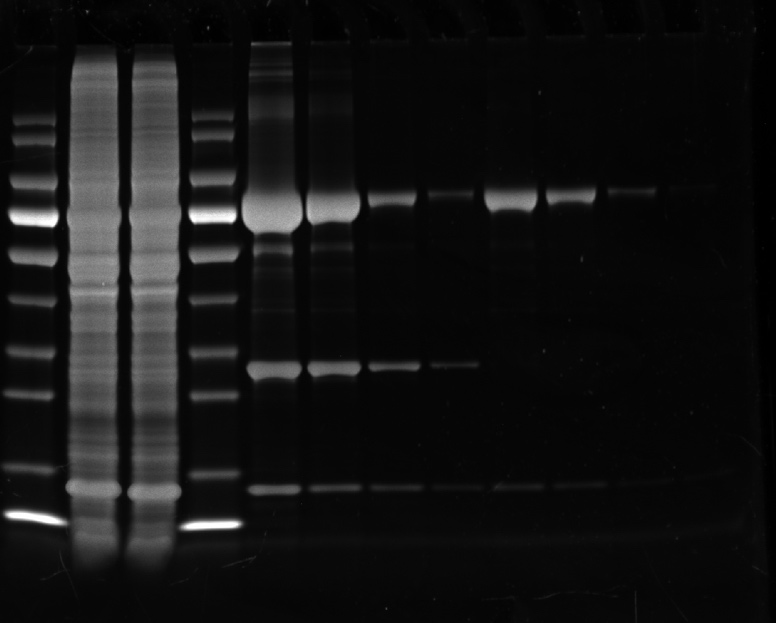
**
